# Effects of Nutritional Conditions on Growth, Biofilm Formation, and Enterotoxin Production in *Staphylococcus aureus* Associated with Food Poisoning

**DOI:** 10.3390/ijms26199791

**Published:** 2025-10-08

**Authors:** Zuo Hu, Zhihao Zhu, Hisaya K. Ono, Shouhei Hirose, Yukiko Hara-Kudo, Shaowen Li, Dong-Liang Hu

**Affiliations:** 1 Department of Zoonoses, Kitasato University School of Veterinary Medicine, Towada 034-8628, Japan; hu.zuo@st.kitasato-u.ac.jp (Z.H.); zhuzhihao@webmail.hzau.edu.cn (Z.Z.); hisaono@vmas.kitasato-u.ac.jp (H.K.O.); 2 College of Veterinary Medicine, Huazhong Agricultural University, Wuhan 430070, China; lishaowen@mail.hzau.edu.cn; 3 Division of Microbiology, National Institute of Health Sciences, Kawasaki 210-9501, Japan; sh-hirose@nihs.go.jp (S.H.); ykudo@hoshi.ac.jp (Y.H.-K.); 4 Laboratory of Microbiology, School of Pharmacy and Pharmaceutical Sciences, Hoshi University, Tokyo 142-8501, Japan

**Keywords:** *Staphylococcus aureus*, staphylococcal enterotoxin, bacterial growth, biofilm formation, nutritional factor, food safety

## Abstract

Staphylococcal food poisoning (SFP) is a common foodborne illness caused by the ingestion of enterotoxins produced by *Staphylococcus aureus*, posing a persistent global public health concern. Although regional differences in implicated food types and predominant enterotoxins have been reported, the underlying factors remain unclear. In this study, we systematically investigated the effects of nutritional factors on the growth, biofilm formation, and production of two representative enterotoxins, SEA and SEB, by *S. aureus*. Specifically, we evaluated bacterial responses to different concentrations of NaCl, glucose, and tryptone. NaCl suppressed growth, biofilm formation and enterotoxin production in a dose-dependent manner. Glucose markedly inhibited both bacteria growth and enterotoxin production, with a stronger effect on SEB than SEA. In contrast, tryptone promoted bacterial growth and moderately enhanced biofilm formation but did not significantly affect enterotoxin production. Importantly, even under comparable bacterial counts, the types and amounts of SEs produced varied substantially depending on the nutrient composition. These findings provide new insights into the nutrient-dependent regulation of virulence in *S. aureus* and highlight the importance of considering environmental and nutritional factors when assessing risks of SFP and designing effective food safety strategies.

## 1. Introduction

Staphylococcal food poisoning (SFP) is caused by the ingestion of preformed staphylococcal enterotoxins (SEs) produced by *S. aureus* in contaminated foods, and represents a significant global concern in terms of public health and food safety across various regions and countries [1,2]. In the United States, the annual number of SFP cases is estimated at approximately 241,140, imposing a great economic burden [3,4]. According to the European Food Safety Authority (EFSA), a total of 2,454 outbreaks of SFP were reported in the European Union in 2015, and 1,400 cases were recorded in 2019 [5,6]. In Asian countries, such as Japan and China, SFP has been reported as a persistent and year-round public health issue [7,8,9,10,11]. Noteworthy examples in Japan include a large-scale outbreak in Osaka involving contaminated skim milk powder, which affected over 14,000 individuals [7]; a widespread outbreak in 2023 linked to boxed lunches produced in Aomori Prefecture, resulting in 554 cases across 29 prefectures [10]; and a 2024 incident in Yokohama caused by eel-based lunch boxes, which led to fatalities [11]. These incidents underscore the substantial impact of SFP not only on human health but also on societal and economic levels. The recurrence and scale of such outbreaks highlight the urgent need for improved understanding of the bacterial growth, SE production and regulation in various food matrices, as well as the development of more effective control measures in food processing and preservation systems. 

SEs, produced during the growth of *S. aureus* in food matrices, are the causative exotoxins responsible for SFP [1,12]. These exotoxins have attracted considerable attention due to their remarkable resistance to environmental stressors such as heat, osmotic pressure, acidic conditions, digestive enzymes, and irradiation. Consequently, SEs can retain their biological and emetic activities even in foods where *S. aureus* cells have been inactivated through heat treatment, posing a significant risk of intoxication [13,14]. Numerous SEs have been identified, including the classical types staphylococcal enterotoxin A (SEA) to SEE, as well as variant new types such as SEG to SElZ, SEl01, SEl02 [2,15,16]. More recently, several novel genes encoding SEls have been identified, including sel26, sel27, sel28, sel29, sel30, sel31, sel32, and sel33. These genes were found in *S. aureus* genome data collected between 1924 and 2016 [1,17,18,19]. To date, a total of 33 SE and staphylococcal enterotoxin-like (SEl) genes have been identified, including nine variant forms currently undergoing epidemiological evaluation and functional characterization [1,20]. Among them, SEA and SEB are particularly noteworthy due to their high production levels and their frequent involvement in foodborne outbreaks [2,21]. SEA encodes in the genetic element of prophage, and SEB encodes in the pathogenetic island (SaPI3) of the chromosome of *S. aureus* [22,23]. Notably, SEA is the most commonly detected toxin in SFP cases, accounting for approximately 80% of reported incidents in Japan. This prevalence underscores its significance and highlights its value as a critical target for food safety research and public health interventions [24].

The types of foods implicated in SFP include protein-rich foods such as meat, eggs, milk, and their processed products, as well as carbohydrate-rich items such as rice, rice-based processed products, pastries, and bread [1,3,7,20,25]. However, the predominant food types associated with SFP outbreaks vary significantly across countries and regions. In Western countries, including Europe and North America, meat, eggs, dairy products, and their derivatives are most frequently identified as the primary sources of SFP [3,4,5,6,25]. In contrast, in Asia, particularly in countries such as Japan and China, carbohydrate-rich foods such as rice, rice-based processed foods and pastries are more commonly implicated in SFP outbreaks [8,9,10,26]. These regional differences in causative food types may be attributed to variations in food nutrient composition, food processing and storage conditions, as well as traditional dietary habits and cultural practices. Despite the frequent occurrence of SFP, the reasons and mechanisms underlying the geographic differences in causative food types, nutrient environments, dietary patterns, and predominant enterotoxin types remain largely unknown.

In this study, we performed a comprehensive and systematic comparative analysis of *S. aureus* growth, biofilm formation, and the production of representative enterotoxins, SEA and SEB, under different nutritional conditions, including varying concentrations of sodium chloride (NaCl), glucose, and tryptone. The results revealed that nutritional environments significantly and differentially influenced bacterial growth, the types of enterotoxins produced, and their production levels. These findings enhance our understanding of how regional dietary patterns and food processing conditions may affect the epidemiological and clinical characteristics of SFP. Moreover, this study provides valuable theoretical and practical insights for establishing region-specific food processing standards, safety monitoring systems and the development of effective food hygiene strategies to prevent *S. aureus*-related food poisoning.

## 2. Results

### 2.1. S. aureus Growth Dynamics Under Different Nutritional Conditions

**Figure 1:**
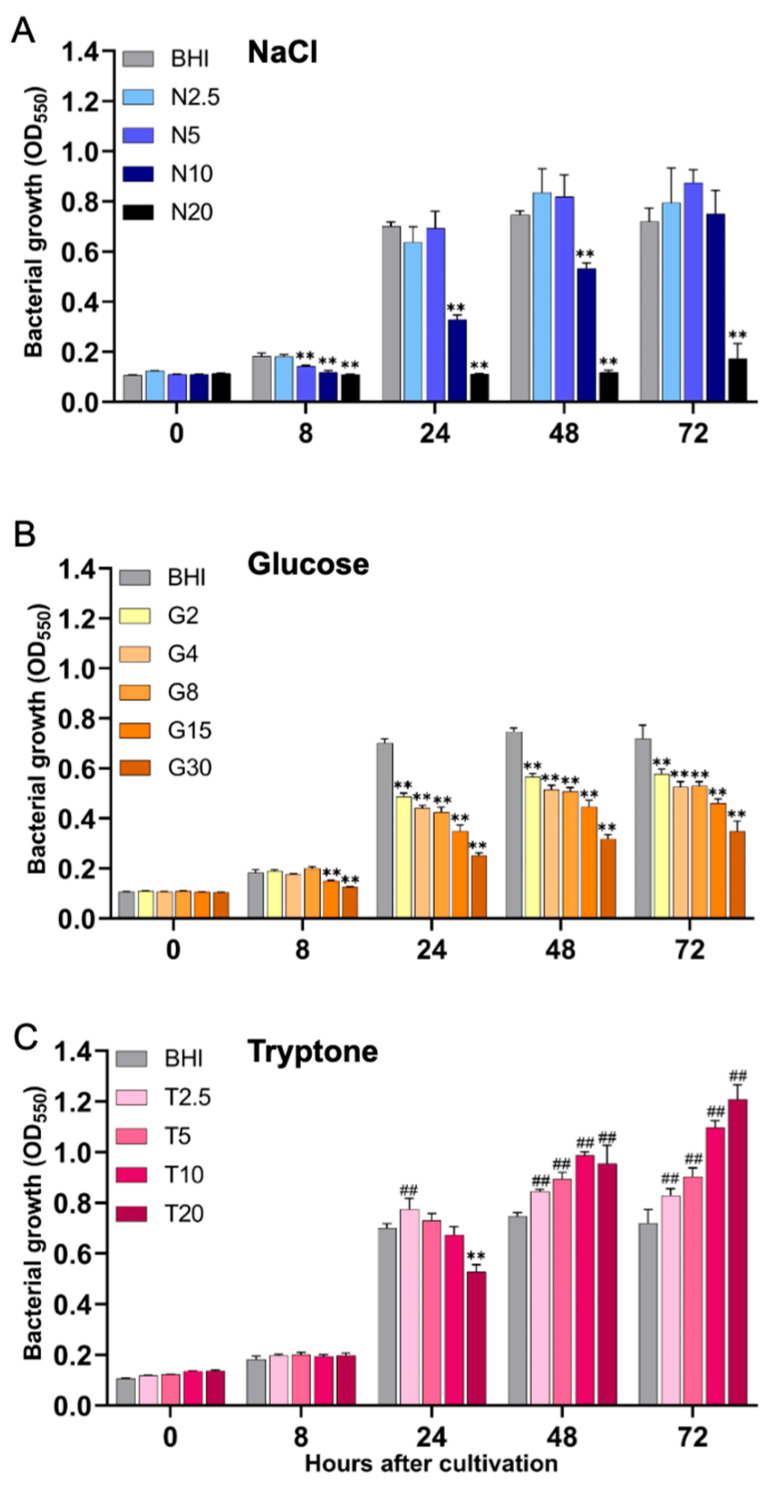
Comparison of bacterial growth characteristics of *S. aureus* FRI-S6 under different nutritional conditions. Bacteria were first cultured in BHI-YE broth for 14 h and subsequently diluted to 6.0 × 10^4^ CFU/mL. In a 96-well flat-bottom microplate, 100 μL of each medium was dispensed into individual wells: BHI-YE alone (negative control), BHI-YE with bacteria (positive control), and BHI-YE supplemented with NaCl (N2.5, N5, N10, N20 = 2.5%, 5%, 10%, 20%) (**A**), glucose (G2, G4, G8, G15, G30 = 2%, 4%, 8%, 15%, 30%) (**B**), or tryptone (T2.5, T5, T10, T20 = 2.5%, 5%, 10%, 20%) (**C**) with bacteria. Each condition was tested in quadruplicate. Plates were incubated statically at 37 °C, and bacterial growth was monitored by OD_550_ at 0, 8, 24, 48, and 72 h. Data are presented as means ± standard deviations based on four to six wells per group at each time point. Statistically significant differences are indicated as ** *p* < 0.01 (lower than the BHI-YE control group); ^##^ *p* < 0.01 (higher than the control group).

In this study, we first conducted a comprehensive and systematic comparative evaluation of the growth dynamics of *S. aureus* under different nutritional environments. Results showed that in the Brain heart infusion supplemented with yeast extract (BHI-YE) control medium, bacterial growth significantly increased over time from 8 to 72 h. In the BHI-YE medium supplemented with lower (2.5 and 5%) concentrations of NaCl, bacterial growth was not markedly affected. However, at the moderate (10%) to high (20%) concentrations of NaCl, both of them significantly inhibited bacterial growth, with the extent of inhibition displaying a clear dose-dependent pattern (Figure 1A). Notably, with prolonged incubation, bacteria gradually adapted to higher concentrations (10%) of NaCl and exhibited rapid growth, reaching levels comparable to the BHI-YE control group at 72 h after cultivation. On the other hand, at a high NaCl concentration (20%), bacterial growth was completely inhibited, showing no significant increase even after 72 h of cultivation. In the BHI-YE media supplemented with 2-30% concentrations of glucose, bacterial growth was markedly suppressed, with the extent of inhibition displaying a clear dose-dependent pattern (Figure 1B). Moreover, under the 30% glucose concentration condition, bacterial growth was more significantly inhibited, showing a 50% reduction, compared with the BHI-YE control condition. In contrast, interestingly, in BHI-YE media supplemented with different concentrations of tryptone, bacterial growth was not inhibited but, rather, was significantly enhanced over a broad concentration range (2.5–20%) (Figure 1C). Additionally, the bacteria growth was observed to significantly increase in a dose-dependent manner at 48 and 72 h post-cultivation.

### 2.2. Biofilm Formation of S. aureus Under Different Nutritional Conditions

**Figure 2:**
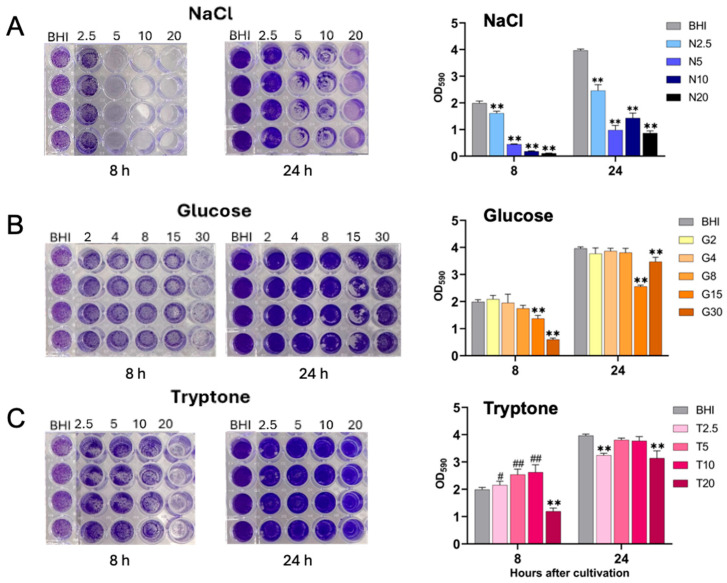
Comparison of biofilm formation by *S. aureus* FRI-S6 under different nutritional conditions. Bacteria were first cultured in BHI-YE broth for 14 h and subsequently diluted to 6.0 × 10^4^ CFU/mL. In a 96-well flat-bottom microplate, 100 μL of each medium was dispensed into individual wells: BHI-YE alone (negative control), BHI-YE with bacteria (positive control), and BHI-YE supplemented with NaCl (N2.5, N5, N10, N20 = 2.5%, 5%, 10%, 20%) (**A**), glucose (G2, G4, G8, G15, G30 = 2%, 4%, 8%, 15%, 30%) (**B**), or tryptone (T2.5, T5, T10, T20 = 2.5%, 5%, 10%, 20%) (**C**). Each condition was tested in quadruplicate. Plates were incubated statically at 37 °C, and biofilm formation was assessed at 8 and 24 h by crystal violet staining. After staining with 0.1% crystal violet for 20 min, excess dye was removed, wells were washed with PBS, air-dried, and photographed. Bound dye was then solubilized with 95% ethanol, and biofilm biomass was quantified by OD_590_. Data are presented as means ± standard deviations based on four to six wells per group at each time point. Statistically significant differences are indicated as ** *p* < 0.01 (lower than the BHI-YE control group); ^#^ *p* < 0.05 and ^##^ *p* < 0.01 (higher than the control group).

We next performed a systematic comparison of *S. aureus* biofilm formation under various nutritional environments, with representative images presented in Figure 2. To quantitatively evaluate biofilm formation, stained biofilms were solubilized with ethanol, and the optical density (OD_590_) was measured using a microplate reader. The results demonstrated that *S. aureus* formed substantial biofilms in BHI-YE control medium after 8 and 24 h of incubation, with significantly enhanced biofilm development observed at 24 h compared to 8 h (Figure 2). In contrast, supplementation with 2.5% NaCl significantly inhibited biofilm formation relative to the BHI-YE control. Moreover, increasing NaCl concentrations led to progressively greater suppression of biofilm formation, indicating a clear dose-dependent inhibitory effect (Figure 2A). Glucose supplementation at concentrations of 2–8% did not exert an inhibitory effect on biofilm formation. However, at higher concentrations (15% and 30%), glucose significantly inhibited biofilm formation at 8 and 24 h post-incubation (Figure 2B). Interestingly, tryptone supplementation at concentrations of 2.5, 5, and 10% significantly promoted biofilm formation at 8 h after cultivation. Conversely, a higher concentration of 20% tryptone resulted in a significant reduction in biofilm formation at 8 and 24 h after cultivation (Figure 2C).

### 2.3. Production of Staphylococcal Enterotoxins, SEA and SEB, Under Different Nutritional Conditions

**Figure 3:**
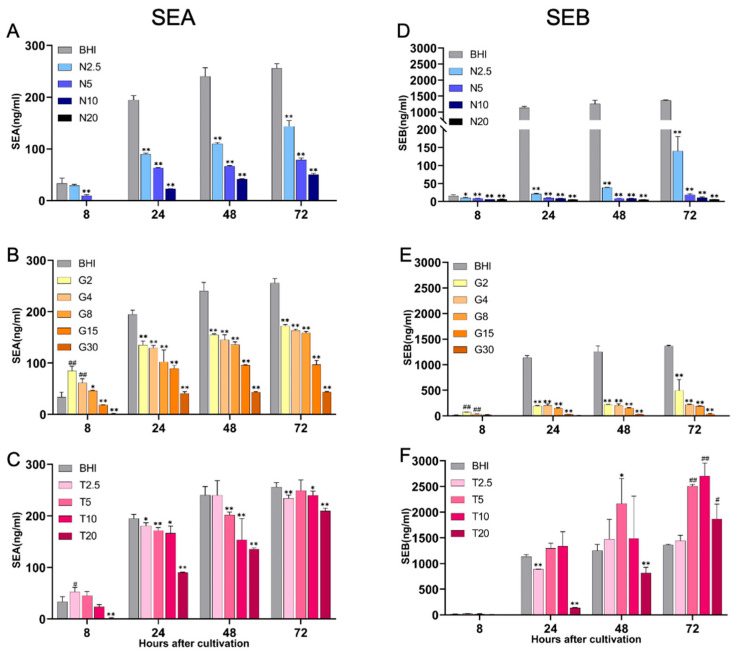
Quantification of staphylococcal enterotoxins SEA and SEB in culture supernatants by sandwich ELISA. Ninety-six–well plates were coated overnight at 4 °C with anti-SEA or anti-SEB antibodies (20 µg/well) and blocked with 1% bovine albumin in PBS. *S. aureus* FRI-S6 was cultured in BHI-YE or BHI-YE supplemented with (**A**,**D**) NaCl (N2.5, N5, N10, N20 = 2.5%, 5%, 10%, 20%), (**B**,**E**) glucose (G2, G4, G8, G15, G30 = 2%, 4%, 8%, 15%, 30%), or (**C**,**F**) tryptone (T2.5, T5, T10, T20 = 2.5%, 5%, 10%, 20%) for 8, 24, 48, and 72 h. Culture supernatants and serially diluted SEA or SEB standards (100–0.78 ng/mL) were added in duplicate and incubated at 37 °C for 1 h. After blocking nonspecific binding with normal rabbit serum, plates were incubated with HRP-conjugated anti-SEA or anti-SEB antibodies (1:1000 or 1:500) for 1 h at 37 °C. Following substrate reaction with OPD/H_2_O_2_ for 20 min in the dark, the reaction was stopped with 1 M H_2_SO_4_. Absorbance was measured at 490 nm, and SEA and SEB concentrations were calculated from standard curves. Data are presented as means ± standard deviations based on four wells per group at each time point. Statistically significant differences are indicated as * *p* < 0.05, ** *p* < 0.01; ^#^ *p* < 0.05 and ^##^ *p* < 0.01.

The production of SEA and SEB under various nutritional conditions was determined and quantified using a previously established sandwich ELISA method [27]. In the BHI-YE control medium, SEA production exhibited a continuous increase from 8 to 72 h post-inoculation (Figure 3, left panel). In contrast, SEA production was significantly inhibited in BHI-YE medium supplemented with NaCl, in a dose-dependent manner, from early exponential to late stationary growth phases when compared with the BHI-YE control (Figure 3A). In BHI-YE medium supplemented with glucose, SEA production was slightly elevated at low glucose concentrations (2% and 4%) at 8 h post-inoculation, corresponding to the early growth phase. However, at later time points (24 to 72 h), SEA production was significantly suppressed at all of the glucose concentrations (2–30%) compared to the BHI-YE control (Figure 3B). In BHI-YE medium supplemented with tryptone, no significant inhibitory effect on SEA production was observed at concentrations ranging from 2.5% to 10%. However, tryptone at a concentration of 20% resulted in a marked reduction in SEA production (Figure 3C). 

We also examined the production of SEB, encoded by the SaPI3 genomic island. SEB production in the BHI-YE control condition also showed a consistent increase from 8 to 72 h post-inoculation (Figure 3, right panel). NaCl and glucose supplementation significantly inhibited SEB production across all tested time points and concentrations (Figure 3D,E). Interestingly, tryptone did not suppress SEB production; rather, SEB levels were significantly increased after 72 h of cultivation in media containing 5% and 10% tryptone (Figure 3F).

### 2.4. Comparison of SE Production Under Different Nutritional Conditions with Comparable Bacterial Cell Densities

**Figure 4:**
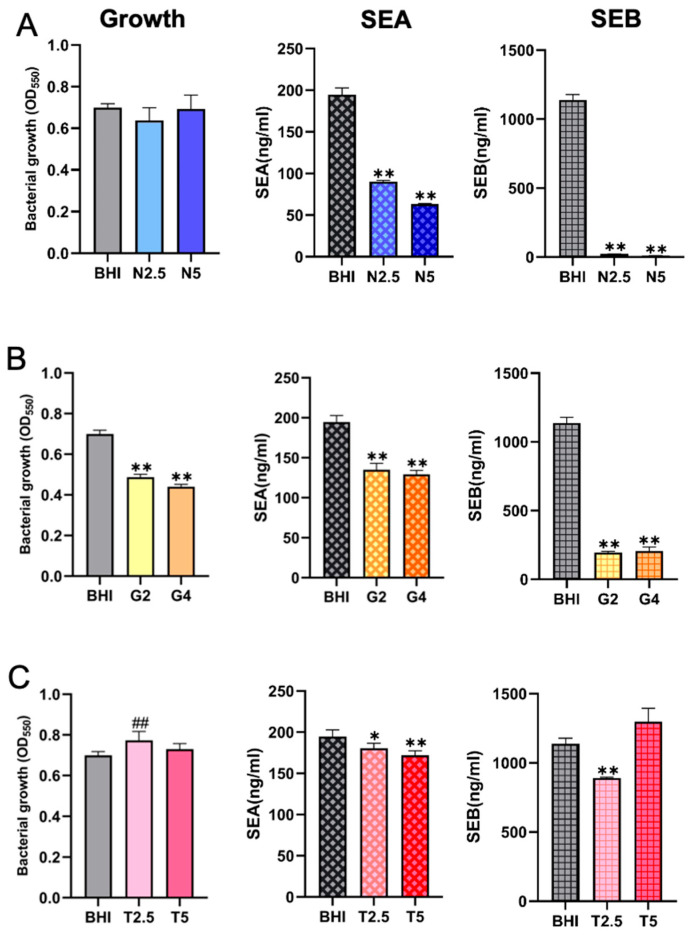
Comparison of staphylococcal enterotoxin (SE) production under different nutritional conditions with comparable bacterial cell densities. *S. aureus* FRI-S6 was cultured in BHI-YE or BHI-YE supplemented with NaCl (N2.5, N5 = 2.5%, 5%), glucose (G2, G4 = 2%, 4%), or tryptone (T2.5, T5 = 2.5%, 5%) for 24 h. Bacterial growth was monitored by OD_550_ to confirm similar cell densities across all conditions. SEA and SEB concentrations in culture supernatants were determined by ELISA. SEA production was significantly reduced in NaCl-supplemented media, while SEB production was almost completely suppressed (**A**). In glucose-supplemented media, both SEA and SEB were markedly decreased compared with the BHI-YE control (**B**). In contrast, tryptone supplementation did not significantly affect SEA or SEB production (**C**). Data are presented as means ± standard deviations. Statistically significant differences are indicated as * *p* < 0.05 and ** *p* < 0.01 (lower than the BHI-YE control group); ^##^ *p* < 0.01 (higher than the control group).

We next compared the production of enterotoxins under different nutritional environments with comparable bacterial cell densities, using BHI-YE control as a reference. To ensure valid comparisons, cultures were grown in BHI-YE or BHI-YE supplemented with NaCl, glucose, or tryptone. After 24 h of cultivation, bacterial growth was monitored by measuring OD_550_, confirming that the final cell densities were nearly identical across all tested conditions. Under standardized growth conditions, SEA production was significantly reduced in media supplemented with 2.5% and 5% NaCl, whereas SEB production was almost completely suppressed relative to the BHI-YE control (Figure 4A). These findings demonstrate a strong inhibitory effect of NaCl on enterotoxin synthesis, which is not strictly correlated with bacterial growth. In media containing 2% and 4% glucose, SEA production parallels growth in BHI-glucose, whereas SEB production is significantly decreased under the same conditions (Figure 4B). Supplementation with 2.5% and 5% tryptone also inhibited SEA production. SEB levels remained comparable to the BHI-YE control, indicating that moderate supplementation with nitrogen sources does not significantly affect SEB production (Figure 4C).

### 2.5. Differential Effects of Nutritional Conditions on the Production of Distinct Enterotoxin Types

**Figure 5:**
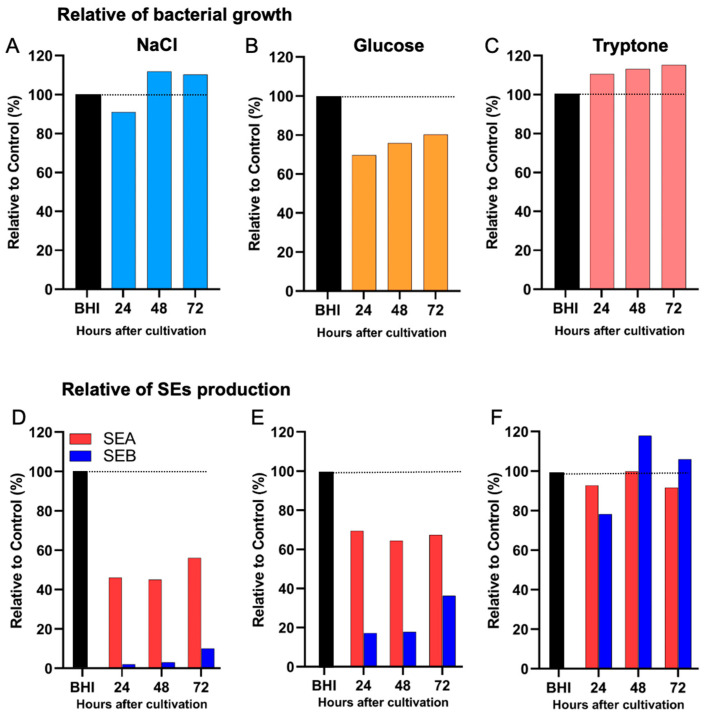
Differential effects of nutritional conditions on the production of distinct enterotoxin types. *S. aureus* FRI-S6 was cultured in BHI-YE or BHI-YE supplemented with (**A**) NaCl (N2.5 = 2.5%), (**B**) glucose (G2 = 2%), or (**C**) tryptone (T2.5 = 2.5%) under conditions yielding comparable bacterial cell densities. Enterotoxin levels in culture supernatants were determined by ELISA at 24, 48, and 72 h. SEA production was reduced to ~40% of the BHI-YE control in NaCl-supplemented medium, while SEB production was almost completely suppressed (5–10% of control) (**D**). In glucose-supplemented medium, SEA levels decreased to ~65% and SEB to ~18% of control (**E**). In contrast, tryptone supplementation did not significantly affect either SEA or SEB production, both remaining comparable to BHI-YE controls across all time points (**F**).

We further examined the differential impact of various nutritional environments on the production of distinct SEs under comparable bacterial cell densities. In the presence of 2.5% NaCl, bacterial growth remained comparable to the BHI-YE control, with nearly identical cell counts observed throughout the 72 h cultivation period. However, under this condition, SEA production was reduced to approximately 40% of the BHI-YE control, whereas SEB production was almost completely suppressed (5–10% of control levels) (Figure 5A), suggesting a much stronger inhibitory effect of NaCl on SEB than on SEA. Similarly, supplementation with 2% glucose markedly inhibited both SEA and SEB production, with SEA levels reduced to 65% and SEB levels to 18% of the BHI-YE control at 24 h (Figure 5B), indicating a disproportionately stronger suppressive effect of glucose on SEB. In contrast, supplementation with 2.5% tryptone did not significantly inhibit either SEA or SEB production, with toxin levels remaining comparable to the BHI-YE control throughout the cultivation period (Figure 5C). These results indicate that, unlike NaCl or glucose, tryptone does not selectively affect SEA or SEB production under the tested conditions.

## 3. Discussion

Staphylococcal food poisoning (SFP), primarily caused by the ingestion of preformed SEs in *S. aureus*-contaminated foods, remains a significant global public health threat. This study provides novel insights into how nutritional environments differentially influence *S. aureus* growth, biofilm formation, and enterotoxin types and production levels. Our results showed that NaCl inhibited biofilm formation at low concentrations and suppressed both growth and the production of both SEA and SEB in a dose-dependent manner. Glucose markedly inhibited bacteria growth and SE production, with stronger suppression of SEB than SEA. Tryptone enhanced bacterial growth and moderately promoted biofilm formation but had no significant impact on SE production. These results demonstrate that certain nutritional factors, specifically NaCl and glucose, can strongly and differentially suppress enterotoxin production, even under conditions where bacterial growth remains largely unaffected. These findings enhance our mechanistic understanding of enterotoxin regulation and also highlight practical implications for developing food safety control strategies in countries with diverse cultural and dietary contexts. 

The bacterial growth analysis revealed that while *S. aureus* growth was markedly inhibited under salt stress, it simultaneously exhibited notable adaptive resilience under salt stress, particularly at NaCl concentrations ≤10% (Figure 1A). During the early phase of salt stress, bacterial growth was suppressed; however, by 72 h of cultivation, proliferative capacity of the bacteria was restored, likely reflecting the activation of osmoprotective mechanisms in *S. aureus*, such as the upregulation of compatible solute transporters (e.g., *pro*T, *put*P) [28,29]. Conversely, our data showed that glucose supplementation consistently and dose-dependently suppressed bacterial growth. These results are consistent with previous reports on glucose-mediated inhibition of staphylococcal proliferation, possibly due to acidification of the culture medium from fermentation byproducts such as lactic acid and/or due to impairment of the unique cell-wall structure, pentaglycine bridges [30,31]. In contrast, tryptone significantly enhanced bacterial growth, suggesting that increased availability of peptides and amino acids positively influences metabolic activity and cellular replication, potentially via CodY-mediated relief of nutrient repression [32].

Biofilm formation, a critical virulence trait in *S. aureus*, was also modulated by the tested nutritional conditions. NaCl and high concentrations of glucose exhibited dose-dependent inhibitory effects on biofilm formation, which may be associated with alterations in the expression of *ica*ADBC, *sar*A, and *agr* loci, previously implicated in biofilm regulation [33,34]. In contrast, low to moderate concentrations of tryptone enhanced biofilm biomass, while excessive tryptone (20%) reduced it, possibly due to nitrogen excess or metabolic imbalances [32]. These findings highlight the complex regulatory interplay between environmental cues and virulence expression, and suggest that the availability of specific nutrients, in conjunction with osmotic stress, may critically shape the pathogenic potential of *S. aureus* in food-related matrices.

A key finding of this study is the differential sensitivity of SEA and SEB production to specific nutritional stressors. Under comparable bacterial densities, NaCl and glucose significantly inhibited SEA production; however, SEB production was much more strongly repressed, by up to 90-95%, suggesting the involvement of toxin-specific regulatory mechanisms. The genetic localization of these toxins may partly explain this phenomenon. SEA is encoded on prophage elements (e.g., ΦSa3ms), which are typically induced under stress conditions via the SOS response, potentially increasing SEA expression even during environmental challenges [35,36]. In contrast, SEB is encoded on the SaPI (vSa1) pathogenicity island, whose activation depends on finely tuned interplay with the agr quorum sensing system and accessory regulators such as *sig*B, *ccp*A, and *cod*Y [37,38].

Glucose-mediated suppression of SEB may be attributed to carbon catabolite repression (CCR) via CcpA, which inhibits virulence factor expression by modulating RNAIII and other *agr*-dependent transcripts in response to high carbohydrate levels [39,40]. Meanwhile, salt stress may impact *sig*B activity or osmotic stress response pathways, which in turn can repress *seb* transcription more stringently than *sea*. These findings align with recent transcriptomic studies indicating that SEB-encoding genes exhibit higher sensitivity to environmental regulatory inputs compared to SEA [35,41]. Moreover, this observation is particularly relevant for understanding regional SFP outbreaks, as carbohydrate-rich foods (e.g., rice, noodles, pastries) are more commonly implicated in Asian countries, while protein-rich foods (e.g., meat, egg, dairy, fish) dominate in Western countries [1,42,43,44].

Interestingly, tryptone supplementation did not suppress either SEA or SEB production. At moderate concentrations (2.5–10%), both toxins were stably expressed at levels comparable to the BHI-YE control. This supports the hypothesis that peptide-rich environments, as found in processed meats or dairy-based foods, may facilitate sustained enterotoxin production. It is possible that tryptone acts not only as a nitrogen source but also as a signaling cue that mitigates CodY-mediated repression of virulence genes [45]. Our data also suggest that neither SEA nor SEB is subject to strong feedback inhibition in amino acid-rich environments, raising concerns about the persistence of toxin activity in protein-rich food matrices even after heat treatment.

Collectively, these findings provide important implications for food hygiene and SFP prevention. Our data indicate that certain food additives, such as sodium chloride and glucose, can selectively modulate enterotoxin synthesis in a toxin-specific manner, independent of their effects on bacterial growth. This highlights the need to consider not only microbial growth but also virulence regulation when evaluating food safety. The production of SEs is not always correlated with bacterial growth or biofilm formation, indicating that bacteria growth-based microbial testing may underestimate the actual toxigenic risk, particularly in foods where stress conditions differentially impact toxin gene expression. The markedly stronger inhibitory effect of NaCl and glucose on SEB production compared to SEA may help explain the epidemiological pattern observed in Japan, where SEA is the most frequently reported toxin in SFP outbreaks, especially those associated with carbohydrate-rich foods such as rice, noodles, and pastries. These findings emphasize the importance of developing region-specific food safety strategies that account for local dietary patterns and the nutritional microenvironment of food matrices. Future studies should explore the underlying molecular mechanisms through transcriptomic and proteomic profiling under varying nutrient conditions and various temperature conditions. Additionally, it would be valuable to validate these findings in actual food matrices, such as rice, meat, and dairy products, and evaluate the impact of processing conditions on SE stability and bioactivity.

## 4. Materials and Methods

### 4.1. Bacterial Strains and Culture Conditions

The bacterial strain FRI-S6, isolated from a food poisoning case, was used in this study. FRI-S6 carries the *sea* gene within the prophage ϕSa3mu, and the *seb*, *sek*, and *seq* genes within the pathogenicity island SaPI (vSa1). The FRI-S6 strain was revived from storage at −80 °C by streaking onto a tryptic soy agar (TSA) plate, followed by incubation at 37 °C overnight to obtain isolated single colonies, which were then used for subsequent growth experiments under different conditions.

### 4.2. Preparation of Different Nutritional Culture Media

Brain heart infusion supplemented with yeast extract (BHI-YE) was used as a control medium. BHI powder (37 g; Eiken Chemical Co., Ltd., Tokyo, Japan) and extra dried yeast extract (10 g; Nacalai Tesque, Inc., Kyoto, Japan) were completely dissolved in 1000 mL of distilled water. The medium was autoclaved at 121 °C for 15 min. NaCl gradient media: Sodium chloride (10 g; Nacalai Tesque, Inc., Kyoto, Japan) was completely dissolved in 40 mL of BHI-YE to prepare a 20% NaCl (the water activity was approximately 0.89) medium (N20). The solution was sterilized using a 0.22 µm filter (Millex-HV, 0.22 µm; Merck, Rahway, NJ, USA) under aseptic conditions. To prepare lower concentrations, 20 mL of the N20 solution was mixed with 20 mL of BHI-YE to obtain a 10% NaCl medium (N10). This dilution procedure was repeated sequentially to obtain 5% (N5) and 2.5% (N2.5) NaCl media. Glucose gradient media: D-(+)-Glucose (15 g; Kanto Chemical Co., Inc., Tokyo, Japan) was completely dissolved in 35 mL of BHI-YE to obtain a 30% glucose (the water activity was approximately 0.97) medium (G30). The solution was sterilized using a 0.22 µm filter under aseptic conditions. To prepare a 15% glucose medium (G15%), 20 mL of G30 was mixed with 20 mL of BHI-YE. This dilution process was further repeated to prepare 8% (G8), 4% (G4), and 2% (G2) glucose media. Tryptone gradient media: Tryptone (10 g; Nacalai Tesque, Inc., Kyoto, Japan) was dissolved in 50 mL of BHI-YE to obtain a 20% tryptone (the water activity was approximately 0.99) medium (T20). After complete dissolution, the solution was sterilized using a 0.22 µm filter under aseptic conditions. To prepare a 10% tryptone medium (T10), 20 mL of T20 was mixed with 20 mL of BHI-YE. This procedure was repeated to obtain 5% (T5) and 2.5% (T2.5) tryptone media. We confirm that each batch of media was checked for sterility. After 0.22 µm filtration, no bacterial growth was observed in our experiments. The nutrient concentrations were established at levels that produced clear effects on bacterial growth and were adjusted by two-fold serial dilutions, rather than being based on concentrations present in foods.

### 4.3. Determination of Bacterial Growth

A single colony of *S. aureus* FRI-S6 was picked and inoculated into a 50 mL culture tube containing 5 mL of BHI-YE medium. The culture was incubated statically at 37 °C for 16 h. The culture was then diluted with BHI-YE to adjust the bacterial concentration to 6 × 10^5^ CFU/mL, and further diluted with the corresponding test media to a final concentration of 6 × 10^4^ CFU/mL. In a 96-well microplate, 100 µL of each medium: BHI-YE alone (negative control), BHI-YE with bacteria (positive control), and bacteria in NaCl-supplemented media (2.5, 5, 10, and 20% were abbreviated as N2.5, N5, N10, and N20, respectively), glucose-supplemented media (2, 4, 8, 15, and 30% were abbreviated as G2, G4, G8, G15, G30, respectively) and tryptone-supplemented media (2.5, 5, 10, and 20% were abbreviated as T2.5, T5, T10, T20, respectively), was dispensed into individual wells, with each condition tested in quadruplicate. The plate was incubated statically at 37 °C. Bacterial growth was monitored by measuring optical density at 550 nm (OD_550_) using a Microplate spectrophotometer (Multiskan SkyHigh, Thermo Scientific, Life Technologies Holdings Pte Ltd., Singapore) at five time points: 0, 8, 24, 48, and 72 h after cultivation.

### 4.4. Detection and Analysis of Biofilm Formation

The preparation of bacterial inoculation and media with various nutritional conditions was carried out following the same procedures as described for bacterial growth determination. Inoculated samples were added to 96-well microtiter plates at 100 μL per well, with 4 to 6 replicate wells per sample. The plates were incubated statically at 37 °C. At 8 and 24 h post-incubation, the culture was stopped, and the bacterial suspension in each well was gently removed. Subsequently, 100 μL of 0.1% crystal violet solution was added to each well for staining for 20 min. The excess dye was then discarded, and the wells were washed twice with PBS. After shielding from light and allowing to air dry, photographs were taken. To quantify biofilm formation, 100 μL of 95% ethanol was added to each well to solubilize the stained biofilm, and the optical density (OD) of the resulting solution was measured at 590 nm using a Microplate spectrophotometer (Multiskan SkyHigh, Thermo Scientific, Life Technologies Holdings Pte Ltd., Singapore) [46].

### 4.5. Determination of Enterotoxin Production by Sandwich ELISA

The concentration of the enterotoxins SEA and SEB in the culture supernatants was determined using a sandwich ELISA [27]. Briefly, 96-well microplates were coated overnight at 4 °C with anti-SEA or anti-SEB antibodies (20 µg per well) diluted in carbonate-bicarbonate buffer. After removal of the coating solution, plates were washed three times with PBS containing 0.05% Tween 20 (PBST) and then blocked with 1% bovine albumin (BA) in PBS at 37 °C for 1 h. Samples and SEA or SEB standards (prepared by serial two-fold dilution from 100 ng/mL to 0.78 ng/mL in 0.1% BA) were added to the wells (100 µL/well) in duplicate and incubated at 37 °C for 1 h. After washing, normal rabbit serum (diluted 1:100 in 0.1% BA) was added to each well to block nonspecific binding by protein A. Plates were then washed and incubated with HRP-conjugated anti-SEA or anti-SEB antibodies (1:1000 or 1:500 in 0.1% BA) at 37 °C for 1 h. Following washing, the substrate solution consisting of ortho-phenylenediamine (OPD) in citrate-phosphate buffer with H_2_O_2_ was added (100 µL/well), and plates were incubated at room temperature in the dark for 20 min. The reaction was stopped by adding 1 M H_2_SO_4_ (100 µL/well), and absorbance was measured at 490 nm using a microplate spectrophotometer (Multiskan SkyHigh, Thermo Scientific, Life Technologies Holdings Pte Ltd., Singapore). SEA and SEB concentrations in samples were calculated based on a standard curve generated from the absorbance of SEA and SEB standards.

### 4.6. Data Analysis

Three independent biological replicates were performed on different dates using identical procedures, and the resulting data were combined for analysis. Statistical analyses were performed using GraphPad Prism version 9.3.0 (GraphPad Software, San Diego, CA, USA). One-way analysis of variance (ANOVA) was used to assess differences among groups. Differences were considered statistically significant at *p* < 0.05. 

## Data Availability

The original contributions presented in the study are included in the article, further inquiries can be directed to the corresponding author.
